# Diagnostic challenges in posterior circulation ischemic stroke presenting with acute dizziness and vertigo

**DOI:** 10.3389/fneur.2026.1960712

**Published:** 2026-09-24

**Authors:** Jessica Seetge, Alexander Andrea Tarnutzer

**Affiliations:** 1 Department of Neurology and Stroke Center, University and University Hospital Basel, Basel, Switzerland; 2 Faculty of Medicine, University of Zurich, Zurich, Switzerland; 3 Department of Neurology, Cantonal Hospital Baden, Baden, Switzerland

**Keywords:** bedside testing, dizziness, misdiagnosis, posterior circulation stroke, vertigo

## Abstract

Posterior circulation stroke (PCS) is a common and clinically important cause of acute dizziness and vertigo, accounting for approximately 20–25% of all acute ischemic strokes. Because PCS frequently presents with non-specific, fluctuating, or isolated vestibular symptoms, it is about three times more likely to be initially missed in emergency care than anterior circulation stroke (ACS), with important consequences for acute treatment, secondary prevention, and patient outcomes. Diagnostic evaluation is further complicated by the low sensitivity of prehospital stroke scales for posterior fossa syndromes, the under-representation of PCS deficits on the National Institutes of Health Stroke Scale (NIHSS), and the limited early sensitivity of non-contrast computed tomography (NCCT) and diffusion-weighted magnetic resonance imaging (DWI-MRI) in the posterior fossa. In contrast, structured bedside assessment can improve recognition when applied by trained clinicians. However, no single diagnostic pathway covers all patients with PCS presenting with acute dizziness and vertigo: the Head Impulse, Nystagmus, and Test of Skew examination (HINTS) and its extension with bedside hearing assessment (HINTS+) require spontaneous or gaze-evoked nystagmus, gait and posture-based approaches require relevant postural instability, and a clinically relevant subgroup presents with neither. This review outlines the main diagnostic challenges along the care pathway and highlights practical opportunities to improve recognition and reduce missed PCS in patients presenting with acute dizziness and vertigo.

## 1 Introduction

Posterior circulation stroke (PCS) accounts for approximately 20–25% of all acute ischemic strokes (1) and is associated with high morbidity, mortality, and risk of recurrence (2, 3). Diagnosis remains difficult in the hyperacute setting because, compared with anterior circulation stroke (ACS), patients with PCS often present late (4, 5) and are more likely to have non-specific or fluctuating symptoms, which puts them at increased risk of being misdiagnosed or missed in the emergency department (ED) (6, 7) and of having worse outcomes (8) due to complications and recurrent events (9) resulting from a lack of secondary stroke prevention.

The aim of this narrative review is to outline the main diagnostic challenges in patients with PCS presenting with acute dizziness and vertigo along the acute care pathway. We first summarize the clinical presentation according to the vascular territory involved, then discuss the extent of and factors contributing to misdiagnosis in emergency care, the limitations of prehospital and in-hospital stroke scales as well as of early neuroimaging, and the diagnostic value and shortcomings of structured bedside assessment. This sequence follows the patient from undifferentiated presentation to the identification of PCS, and the guiding argument throughout is that no single diagnostic pathway covers all patients with PCS presenting with these symptoms. Based on this, we highlight practical opportunities to improve recognition and identify areas where the diagnostic gap remains unresolved.

For this narrative review, a non-systematic literature search was performed in PubMed for publications up to June 30, 2026, combining terms related to the stroke entity and vascular territory, the clinical presentation, the diagnostic setting and diagnostic errors, the bedside and imaging approaches of interest, and acute treatment and outcome. The full list of search terms, grouped by topic, is provided in Supplementary material. Reference lists of relevant publications were screened for additional literature, publications in English and German were considered, and publications were selected on the basis of their relevance to the clinical questions addressed here. Original research articles were the primary source, complemented by systematic reviews and meta-analyses where pooled estimates were available and by clinical practice guidelines where relevant. Given the heterogeneity of the available literature with respect to study design, patient populations, and reference standards, no formal systematic review methodology according to the PRISMA guidelines was applied. Where estimates conflicted, pooled estimates from systematic reviews and meta-analyses were preferred over single-cohort results and the population and study design underlying each estimate are reported alongside it.

## 2 Clinical presentation

PCS may involve the brainstem, cerebellum, thalamus, or the occipital cortex (10). Depending on the structures affected, this may impair the vestibular and ocular motor networks, the cerebellar circuits involved in balance and coordination, and/or the visual pathways. PCS can therefore present with a variety of symptoms and signs, many of which overlap with those of ACS or stroke mimics (11–13). Dizziness and vertigo are among the most common and clinically challenging symptoms, and may occur in isolation or alongside symptoms including headache, nausea and vomiting, visual disturbance, sensory symptoms, or confusion. Clinical signs may include unilateral limb weakness, dysarthria, gait or truncal ataxia, visual field loss, (subtle) ocular motor abnormalities including nystagmus, altered consciousness or other focal neurological deficits (11). In a recent systematic review and meta-analysis, the most frequent symptoms in PCS were dizziness and vertigo and nausea and vomiting, each reported in approximately 41% of patients, while limb weakness and dysarthria were the most frequent clinical signs, each present in approximately 48% (14).

The clinical syndrome depends largely on the vascular territory affected (Table 1). Posterior inferior cerebellar artery (PICA) strokes are among the most common vascular causes of dizziness and vertigo (15), and infarction of the medial PICA territory most often presents with isolated dizziness or vertigo (defined as dizziness or vertigo without additional obvious central findings such as limb weakness or dysarthria) (16, 17). Involvement of the lateral PICA territory, in contrast, typically produces crossed brainstem findings (18). Anterior inferior cerebellar artery (AICA) infarction should be suspected when dizziness or vertigo is accompanied by unilateral hearing loss (19, 20).

**Table 1:** Affected anatomical structures and typical clinical findings by vascular territory in patients with posterior circulation stroke (PCS) presenting with acute dizziness and vertigo.

Vascular territory	Structures involved	Vestibular and ocular motor findings	Associated clinical findings
SCA	Superior cerebellar peduncle, dorsal (oculomotor) vermis, anterior lobe of the cerebellum, rostral pons (115)	Nystagmus in 27%: spontaneous nystagmus, predominantly horizontal, ipsilesional (24%), and direction-changing gaze-evoked nystagmus (12%) (115, 116); saccadic contrapulsion and bilateral horizontal pursuit impairment (superior cerebellar peduncle) (109, 117); hypometric saccades (dorsal oculomotor vermis) (112)	Limb and gait ataxia, dysarthria, truncal instability (115)
PICA, medial branch	Cerebellar nodulus, ventral uvula, cerebellar tonsil (dorsal paraflocculus) (118, 119)	Isolated dizziness or vertigo, mimicking AUVP with isolated nodulus infarction (118); spontaneous nystagmus, ipsilesional (119); periodic alternating nystagmus, apogeotropic horizontal positional nystagmus, and impaired downward smooth pursuit (nodulus, ventral uvula) (109); direction-changing gaze-evoked nystagmus, geotropic horizontal positional nystagmus, gaze-holding deficits, and asymmetric pursuit impairment, worse ipsilesionally (cerebellar tonsil) (16, 17, 109, 119); skew deviation with ipsilesional hypertropia (109); normal h-HIT (bilaterally) (79)	Truncal and gait instability, often without limb weakness or dysarthria (16, 17)
PICA, lateral branch	Dorsolateral medulla, including the vestibular nuclei, nucleus prepositus hypoglossi, inferior cerebellar peduncle, spinal trigeminal nucleus, spinothalamic tract, descending sympathetic tract, and nucleus ambiguus (15, 18, 120)	Spontaneous nystagmus, horizontal-torsional, contralesional (117); horizontal gaze-evoked nystagmus (nucleus prepositus hypoglossi) (117); impaired VOR, ipsilesional (vestibular nuclei) (109); OLD with ipsilesional hypermetric and contralesional hypometric saccades, and contralesional pursuit impairment (inferior cerebellar peduncle) (109–111); ipsiversive ocular tilt reaction (head tilt, skew deviation with contralesional hypertropia, ocular torsion) (109, 121); ocular motility otherwise full, without ophthalmoplegia (117)	Crossed sensory deficit, Horner syndrome, dysphagia, hoarseness, ipsilateral limb ataxia (18, 120)
AICA	Labyrinth, vestibulocochlear nerve and its root entry zone, cochlear nuclei, lateral pons, middle cerebellar peduncle, flocculus and ventral paraflocculus (19, 20)	Spontaneous nystagmus, horizontal contralesional (labyrinth, vestibulocochlear nerve) or ipsilesional (flocculus) (19, 109); pathological h-HIT due to central lesions interrupting the VOR either unilaterally, ipsilesional (labyrinth, vestibulocochlear nerve) or contralesional (flocculus), or bilaterally (20, 85, 109); gaze-evoked and downbeat nystagmus (flocculus) (109); pursuit impairment, asymmetric and worse ipsilesionally (flocculus) or bilaterally severe (middle cerebellar peduncle) (109)	New-onset unilateral hearing loss, tinnitus, facial palsy; combined audiovestibular impairment in 60%, selective vestibular impairment in 5%, selective auditory impairment in 4% (19, 20)

AICA, anterior inferior cerebellar artery; AUVP, acute unilateral vestibulopathy; h-HIT, horizontal head impulse test; OLD, ocular lateral deviation; PICA, posterior inferior cerebellar artery; SCA, superior cerebellar artery; VOR, vestibulo-ocular reflex.

## 3 Misdiagnosis in emergency care

Acute dizziness and vertigo are common in emergency care (21), accounting for approximately 3.45% of all ED visits (22), and are most often caused by less-sinister vestibular (23) or non-neurological conditions (24). Dizziness and vertigo are also one of the most frequent stroke-mimic presentations in the ED, with peripheral vestibular dysfunction as the single most common stroke mimic overall, accounting for 23.2% of all mimics (25). Stroke accounts for 3.2–5.5% of acute dizziness and vertigo presentations (24, 26–28) and 0.7–13.9% of isolated dizziness and vertigo cases (26, 28). At the same time, dizziness and vertigo occur in 47–75% of patients with PCS (11, 29), with isolated dizziness and vertigo as the presenting symptom in 12–25% of cases (17, 30–32). In the same meta-analysis of consecutive, unselected ED patients, in whom neither symptom duration was restricted nor certain (e.g., peripheral-vestibular, non-stroke central-vestibular) disorders were excluded, stroke prevalences in the subgroup of studies in which all patients underwent magnetic resonance imaging (MRI) were 24% for dizziness and vertigo and 19.5% for isolated dizziness and vertigo (28). Although most dizziness and vertigo presentations are not caused by stroke, follow-up studies suggest that these patients may still represent a clinically relevant risk group. Patients discharged from the ED with a diagnosis of dizziness or vertigo had a two-fold higher risk of stroke or cardiovascular events over 3 years of follow-up compared with patients without dizziness or vertigo (33), and patients hospitalized with isolated dizziness or vertigo had a 3.01-fold higher risk of stroke over 4 years than the general population (34).

Reported proportions of acute ischemic strokes missed in the emergency setting range from 20 to 60%. This wide range reflects differences in the definition of a missed stroke, from any initially incorrect working diagnosis to a missed cerebrovascular event at ED discharge, as well as differences in study populations and settings (35, 36). The risk is highest when symptoms are mild, non-specific, or transient (36). In a two-center retrospective cohort of 465 patients with confirmed ischemic stroke, in which a missed stroke was defined as an initially incorrect diagnosis in the ED, 37% of PCS were initially misdiagnosed compared with 16% of ACS, corresponding to an approximately three-fold increase in the odds of a missed diagnosis (35). One review reported that dizziness and vertigo are among the most commonly misdiagnosed stroke presentations (37), with dizziness or vertigo as the main complaint associated with a 14-fold increase in the odds of misdiagnosis compared with motor symptoms, and isolated dizziness missed initially in approximately 50% of cases (26). In retrospective studies from Switzerland and Germany, 4–6% of initially benign vestibular diagnoses were ultimately deemed wrong, including missed ischemic strokes (23, 38). In a separate population-based study of patients aged over 44 years presenting to the ED with dizziness, vertigo or imbalance, 35% of cerebrovascular events were initially misdiagnosed, rising to 44% in those presenting with these symptoms in isolation (26). A comparable proportion was reported in a prospective study of 115 patients with cerebellar infarction, of whom 34% were not recognized at initial presentation, most commonly being diagnosed as peripheral vestibulopathy. Misdiagnosis was associated with nausea and vomiting (odds ratio [OR] 2.3, 95% confidence interval [CI] 1.01–5.5) and with the absence of neurological signs as determined by emergency physicians (OR 3.5, 95% CI 1.5–8.0) (39). In a population-based cohort of patients discharged from the ED with a diagnosis of peripheral vertigo, the relative risk of stroke within the first 7 days was about 50 times higher than in matched controls (40), and among patients with missed cerebellar stroke, permanent morbidity and mortality have been reported at 33 and 40%, respectively (41). Overall, it has been estimated that approximately 15,000–25,000 patients annually suffer serious and potentially preventable harm from missed strokes presenting with dizziness and vertigo (42).

## 4 Limitations of stroke scales and neuroimaging

Improving recognition of PCS requires addressing diagnostic gaps at every stage of care (Table 2), beginning in the prehospital setting, where the accuracy of stroke screening tools varies considerably, in part because of the diverse and often non-specific nature of PCS presentations (43). Scales such as FAST (Face, Arms, Speech, Time) are weighted toward anterior circulation syndromes, do not include dizziness and vertigo or other posterior circulation symptoms, and may miss approximately half of all PCS (44, 45). Extensions such as BE-FAST, adding balance and visual symptoms, reduced the proportion of missed strokes in a retrospective analysis (46), but the benefit was not confirmed prospectively (47). FAST4D, which adds diplopic images, visual field deficits, dizziness/vertigo and dysmetria/ataxia, increased sensitivity for PCS from 55.7 to 95% compared with FAST, at the cost of overtriage (48).

**Table 2:** Overview of stroke scales and clinical risk scores proposed for the recognition of posterior circulation stroke (PCS) in patients presenting with acute dizziness and vertigo.

Score/scale	General clinical elements included	Specific elements tested	Evaluated application	Sensitivity/specificity^*^ (target condition)	Number of studies available, validation study available (yes/no)	Advantages/disadvantages
FAST (44, 45)	Obvious focal neurologic signs	Face, arms, speech, time	Prehospital stroke screening	55.7%/NA for PCS (48)	Large number of studies, validation available	Widely implemented and quick to apply/weighted toward anterior circulation and misses approximately half of all PCS
BE-FAST (46, 47)	Obvious focal neurologic signs	FAST items plus balance (gait imbalance or leg weakness) and eyes (visual symptoms)	Prehospital stroke screening	NA; proportion of missed strokes reduced from 14.1 to 4.4% (all strokes) (46)	Two studies, prospective validation inconsistent	Simple extension requiring no additional equipment/ benefit not confirmed prospectively
FAST4D (48)	Obvious focal neurologic signs, presenting sx	FAST items plus diplopic images, deficit in field of vision, dizziness/vertigo, dysmetria/ataxia	Prehospital stroke screening	95%/NA for PCS (48)	Single prospective study, no external validation	Highest sensitivity for PCS among prehospital scales/ substantial overtriage, with 224 of 434 false positives attributable solely to the 4D items
Sudbury Vertigo Risk Score (49)	Presenting sx, vascular risk factors, obvious focal neurologic signs	Structured risk items for serious causes	Acute vertigo or dizziness in the ED	For a cut-off value of <5: 100%/69.2% for serious causes (49)	Derivation with validation study available	Reliably identifies low-risk patients (post-test probability <0.5%)/rules out serious causes in general rather than identifying PCS
DEFENSIVE (50)	Presenting sx	Disequilibrium, floating sensation, non-specific dizziness, imbalance, vertigo	Dizziness-related presentation in the ED	100%/NA for PCS (50)	Single study, no external validation	Developed specifically for PCS recognition/specificity not reported and external validation limited
NIHSS (3, 51, 53)	Obvious focal neurologic signs	Standard 11-item scale	Stroke severity assessment	NA	Extensively validated for severity, not for PCS detection	Universally established and reproducible/posterior deficits underrepresented, and a score of 0 does not exclude stroke
e-NIHSS (54)	Obvious focal neurologic signs	NIHSS with nystagmus added to the eye movement item, cranial nerve IX and XII deficits and Horner syndrome to the facial palsy item, and truncal ataxia to the limb ataxia item	Stroke severity assessment	NA	Single study, limited validation	Better reflects posterior circulation deficits with scores 2 points higher than NIHSS in PCS/addresses severity and prognosis rather than early recognition
IVBSS (55)	Obvious focal neurologic signs	11 items including dysphagia and gait ataxia, weighted by clinical experience	Stroke severity assessment	NA	Single study, limited validation	Developed specifically for vertebrobasilar stroke, with excellent inter-rater agreement for dysphagia and gait ataxia/rarely used in routine ED assessment
Posterior NIHSS (56)	Obvious focal neurologic signs	NIHSS plus dysphagia (4 points), abnormal cough (5 points), gait or truncal ataxia (3 points)	Prognosis in mild-to-moderate PCS	NA	Single study, not widely validated	Improves prognostic accuracy in mild-to-moderate PCS/not designed for detection

^*^Whenever available, data from systematic reviews and meta-analyses were reported. BE-FAST, Balance, Eyes, Face, Arm, Speech, Time; DEFENSIVE, DisEquilibrium, Floating sEnsation, Non-Specific dizziness, Imbalance, and VErtigo; ED, emergency department; e-NIHSS, expanded National Institutes of Health Stroke Scale; FAST, Face, Arms, Speech, Time; FAST4D, FAST extended with Diplopic images, Deficit in the field of vision, Dizziness/vertigo, and Dysmetria/ataxia; IVBSS, Israeli Vertebrobasilar Stroke Scale; NA, not available; NIHSS, National Institutes of Health Stroke Scale; PCS, posterior circulation stroke; sx, symptoms.

Within the ED, more targeted tools have been proposed for patients presenting with dizziness and vertigo. The recently validated Sudbury Vertigo Risk Score supports ED risk stratification by identifying patients at low risk for serious diagnoses, defined as stroke, transient ischemic attack (TIA), vertebral artery dissection, or brain tumor. Using a score <5 to define low risk, it showed 100% sensitivity and 69.2% specificity, corresponding to a post-test probability below 0.5%. However, its purpose is to rule out serious diagnoses rather than to identify PCS specifically (49). Another proposed tool, the DEFENSIVE (DisEquilibrium, Floating sEnsation, Non-Specific dizziness, Imbalance, and Vertigo) stroke scale, was developed to improve recognition of PCS in ED patients with dizziness-related symptoms. Although the DEFENSIVE stroke scale reported 100% sensitivity and a reduction in misdiagnosis from 5.2 to 0%, these findings should be interpreted with caution, as external validation in larger ED populations remains limited (50).

Even when PCS is recognized, the National Institutes of Health Stroke Scale (NIHSS) often underestimates its severity and prognostic impact (3). The NIHSS focuses primarily on anterior circulation deficits, while posterior circulation symptoms and signs are underrepresented or absent. NIHSS scores of 4 or below are reported in 62.9% of patients with PCS compared with 34.8% of those with ACS (51, 52), and a score of 0 does not exclude stroke (53). Several posterior-circulation-specific modifications have been proposed to address this limitation, including the expanded NIHSS (e-NIHSS) (54) and the Israeli Vertebrobasilar Stroke Scale (IVBSS) (55). The posterior NIHSS was developed to improve prognostic accuracy in mild-to-moderate PCS by adding dysphagia (4 points), abnormal cough (5 points), and gait or truncal ataxia (3 points) to the standard scale (56). However, these tools mainly address severity and prognosis rather than early recognition of PCS, have not been widely validated across different clinical settings, and their broader adoption in routine ED assessment remains limited.

Imaging also has important limitations in PCS. Non-contrast computed tomography (NCCT) has low sensitivity for detecting acute ischemic stroke in the posterior fossa (57), with reported sensitivity for ischemic changes of only up to approximately 40% (58). Sensitivity is even lower in patients presenting with dizziness or vertigo, ranging from 7 to 28.5% (59, 60). NCCT retains a defined role in this population. In more than one third of patients with acute PCS, a hyperdense vessel sign may be the only computed tomography (CT) finding (61). Despite these limitations, approximately 50% of ED patients presenting with dizziness or vertigo undergo CT as the primary imaging modality (62, 63). Recent data suggest that CT perfusion (CTP) may provide additional diagnostic value when combined with NCCT for detecting acute PCS, depending on lesion location, vascular territory and acquisition protocol (64, 65). Diffusion-weighted MRI (DWI-MRI) remains the most sensitive routine imaging modality for detecting acute ischemia (66), with a reported sensitivity of 79.8% in patients presenting with acute dizziness or vertigo (60). Even so, false-negative findings remain an important limitation, and the probability of a false-negative scan depends on lesion size and location, the interval between symptom onset and imaging, and the imaging protocol. PCS is about five times more likely than ACS to have an initially negative DWI scan (67). False-negative initial DWI-MRI has been reported in 17.6% of patients with isolated vestibular syndrome due to PCS (17), in approximately one in five PCS imaged within the first 24–48 h after symptom onset (42), and in up to 53% of infarcts ≤10 mm (68). Each imaging modality answers different questions and has different blind spots.

Vascular imaging deserves separate consideration. In selected patients, in particular younger patients, patients with neck pain or recent cervical trauma and patients with recurrent stereotyped episodes, evaluation of the vertebral and basilar arteries with CT or MRI angiography should complement parenchymal imaging, since vertebral artery dissection and vertebrobasilar stenosis or occlusion are relevant causes of PCS and change acute management and secondary prevention. Ultrasound of the vertebral arteries may serve as a non-invasive adjunct where angiographic imaging is not readily available (69).

## 5 Bedside neurological examination

Bedside findings in acute dizziness and vertigo are best understood as modifiers of pretest probability. The probability of PCS before examination is set by the presentation and its context (Section 3), the examination raises or lowers it and the resulting post-test probability determines whether imaging, observation or discharge is appropriate. Where the cited meta-analyses allow, likelihood ratios are therefore reported below alongside sensitivity and specificity values.

Evidence-informed recommendations and clinical practice guidelines for the emergency evaluation of acute dizziness and vertigo exist, most prominently GRACE-3 (70). However, they are based on expert consensus rather than randomized evidence, are not universally implemented and rest on a heterogeneous evidence base. One widely proposed approach is ATTEST (Associated symptoms, Timing, Triggers, Examination Signs, and Testing) (71), formerly TiTrATE (Timing, Triggers, and Targeted Examination) (72), which emphasizes associated symptoms, timing and triggers and targeted examination rather than symptom quality (73). This approach is clinically important because patients’ subjective description of dizziness type (“What do you mean by ‘dizzy’?”) has long dominated the clinical paradigm (74), despite evidence that it is diagnostically unreliable (71). Although international consensus definitions distinguish dizziness as “the sensation of disturbed or impaired spatial orientation without a false or distorted sense of motion,” from vertigo as “the sensation of self-motion when no self-motion is occurring or the sensation of distorted self-motion during an otherwise normal head movement” (75). This distinction has little diagnostic value in routine clinical practice, and patients rarely use these terms consistently (12). In a cross-sectional study by Newman-Toker et al., more than half of ED patients changed their description of dizziness when the same question was repeated 5–10 min later (76). When asked to choose from six symptom descriptors, patients selected an average of 2.6, and 75% of those who initially chose something other than vertigo nevertheless described symptoms consistent with vertigo when questioned more specifically (76). Despite this, approximately 64% of emergency physicians ranked symptom type as the most important symptom attribute, and 69% reported basing clinical decisions on it, for example by not pursuing cardiovascular causes when a patient described vertigo (77). A comparable pattern was observed in a Swiss national survey of 152 primary care physicians, in which 92% strongly agreed that asking about the type of dizziness is important, whereas agreement was lower for the frequency (72%) and duration (70%) of attacks (78).

Patients with PCS causing acute dizziness or vertigo most commonly present with an “acute vestibular syndrome” (AVS), defined as acute onset of persistent, continuous dizziness or vertigo (lasting longer than 24 h) in association with nausea or vomiting, head-motion intolerance, nystagmus and gait instability (21). PCS accounts for approximately 25% ± 15% of AVS cases (21, 22, 38, 79), with acute unilateral vestibulopathy (AUVP) as the main peripheral differential diagnosis (71, 80). Although PCS represents only a minority of all AVS presentations, it accounts for the majority of central AVS cases (79%), whereas other central causes, including multiple sclerosis (MS), are less common (11%) (21). This is clinically relevant because dizziness and vertigo are common in MS overall (15%) (81), but isolated acute dizziness or vertigo appears to be an uncommon MS presentation. In one study of 75 patients with a clinically isolated MS-like syndrome involving the brainstem or cerebellum, vertigo was reported in only 19% (82). Thus, while MS remains an important central cause of AVS, the main diagnostic challenge in AVS is usually distinguishing PCS from AUVP.

### 5.1 Head impulse, nystagmus, and test of skew (HINTS) examination

The Head Impulse, Nystagmus, and Test of Skew (HINTS) examination is the most established bedside tool for distinguishing peripheral from central causes of AVS. It consists of three components: assessment of nystagmus type, the horizontal head impulse test (h-HIT), and assessment of skew deviation with alternate cover testing. HINTS+ extends the examination by adding bedside hearing assessment for the detection of AICA-territory strokes (83). In patients with AVS who present with spontaneous or gaze-evoked nystagmus (i.e., “nystagmus that is present with the patient looking straight ahead or to either side but not moving their head” (84). HINTS and HINTS+ performed by at least moderately trained examiners (85, 86) have high diagnostic accuracy and may outperform early DWI-MRI (87). In routine practice, however, only a minority of emergency physicians use these bedside tests, and even fewer report confidence in performing or interpreting them (88). Several groups have also studied video head impulse testing (vHIT), which quantifies vestibulo-ocular reflex (VOR) gain, to differentiate PCS from AUVP (89–91). In a recent meta-analysis, vHIT distinguished stroke from AUVP with a positive likelihood ratio of 5.6 and a negative likelihood ratio of 0.18 (91). In a prospective single-center study of 46 patients with AVS, video-oculography HINTS (vHINTS), which quantifies all three components of the examination, identified central causes with an accuracy of 94.2%, compared with 88.3% for clinical HINTS performed by experts (92). Only about half of patients presenting with dizziness or vertigo have spontaneous nystagmus (93). Nikles et al. reported that only 50% of stroke patients presenting with dizziness or vertigo had nystagmus (94). Other studies show that 17–34.5% of patients with PCS presenting with dizziness or vertigo have no nystagmus (95, 96). In one study of 35 patients with PCS and an initially negative MRI, HINTS could be applied to only 40% of patients because spontaneous nystagmus was absent at the time of assessment (97). For these patients, the term “acute imbalance syndrome” (AIS) has been proposed, defined as acute onset of persistent, continuous dizziness or vertigo (lasting longer than 24 h) in association with nausea or vomiting, head-motion intolerance, and gait unsteadiness, but without nystagmus (57, 98, 99). AIS is an operational construct rather than a validated clinical entity and the reproducibility of its definition has not been formally studied. It has nonetheless been applied across independent groups for several years (57, 100, 101), and a recent systematic review and meta-analysis found that almost half of central AVS cases presented without nystagmus (99), with current classification work framing AVS as occurring with or without nystagmus. The absence of nystagmus is an examination finding and may depend on visual fixation, the timing of the examination, examiner expertise and vestibular-suppressant medication, and techniques that reduce fixation may reclassify individual patients. Because patients with AIS fall outside the HINTS-based algorithm, bedside assessment relies more heavily on gait and truncal stability or on algorithms such as STANDING (see next section) that assess postural stability as a separate step, and therefore remain applicable in the absence of nystagmus (102).

### 5.2 Gait and postural stability assessment

Standing balance should be assessed systematically in all patients with acute dizziness and vertigo, including both AVS and AIS, because severe postural instability is often under-recognized in both syndromes. However, difficulty standing or walking is not specific for a central cause. In one AVS cohort, gait disturbance was present in all patients, and 50% of both PCS and AUVP patients were unable to stand unassisted (103). A commonly used bedside approach is the graded gait and truncal instability (GTI) rating. In this grading system, grade 1 indicates mild to moderate imbalance while walking independently, grade 2 indicates inability to walk without support, and grade 3 indicates falling in the upright posture (16). Using a similar grading approach, Carmona et al. assessed acute truncal ataxia (ATA) in patients with AVS and showed that grade 2–3 ataxia had 100% sensitivity for PCS, but only 61.1% specificity, while grade 3 ataxia alone was highly specific for PCS, occurring in 20 of 32 PICA strokes and 8 of 10 AICA strokes, but in no patients with AUVP (104). Consistent with this, a recent meta-analysis of 18 studies including 1,025 patients with AVS found that grade 2–3 GTI had moderate diagnostic accuracy for central causes, with a sensitivity of 70.8% and specificity of 82.7% (positive likelihood ratio 4.1, negative likelihood ratio 0.35), whereas grade 3 GTI was highly specific but insufficiently sensitive, with a specificity of 99.1% and sensitivity of 44.0% (positive likelihood ratio 49) (105).

Estimates from the gait/truncal instability and the STANDING literature concern central causes of acute dizziness and vertigo in general. PCS accounts for the majority of central AVS (79%) (21), but central causes also include hemorrhage, demyelination, drug intoxication, thiamine deficiency and tumors. Such targeted balance testing may also be incorporated in structured algorithms. STANDING (SponTAneous Nystagmus, Direction, head Impulse test, standiNG) is a four-step bedside approach that identified central causes of acute dizziness and vertigo with a sensitivity of 100% and a specificity of 94.3% in the original single-center study, in which 98 of 292 patients were assessed with the algorithm (102). In the recent multicenter STANDING-M study, including 456 patients across one academic hub and three community EDs, sensitivity for central causes was 88.2% and specificity 91.6%, resulting in a negative predictive value of 99%, with comparable accuracy across sites (106). More recently, the TiTrATE-STANDING Adapted algorithm combined elements of TiTrATE, HINTS/HINTS+, and STANDING to support ED triage of acute dizziness and vertigo. In a validation cohort of 67 patients at two specialized centers, it showed a sensitivity of 90% but a specificity of only 57.9%, with an overall diagnostic accuracy of 62.7%. Importantly, these preliminary findings await confirmation in larger, unselected ED populations (107). Central causes may also present with clinically relevant ATA in the absence of nystagmus. In a study of patients with acute dizziness or vertigo, ATA without nystagmus was found in 15% of patients, of whom 71.4% had PCS, including 50% with cerebellar stroke (108). Since isolated dizziness or vertigo is one of the most common presenting symptoms of cerebellar stroke (16), and nystagmus is absent in about 50% of patients with cerebellar infarction (9, 93), careful assessment of standing, gait, and truncal stability is especially important when cerebellar stroke is a diagnostic consideration.

However, gait and truncal assessment alone do not fully close the diagnostic gap. A clinically important subgroup of patients with PCS presents with neither spontaneous nystagmus nor marked gait or truncal instability. Among 34 patients with PCS presenting with isolated dizziness or vertigo, most with PICA-territory infarcts (27/34, 79.4%), nystagmus was present in only 32% and postural imbalance in 44%, with only two patients showing severe postural imbalance (17). Similarly, in one study of 35 patients with PCS presenting with isolated dizziness or vertigo and initially negative DWI-MRI, 12 presented without spontaneous nystagmus, and only 6 of them had profound imbalance as the only sign suggesting stroke (97). In patients with isolated dizziness or vertigo, the absence of spontaneous nystagmus or severe imbalance should not be taken as evidence of a peripheral cause (22). This evidence derives from small, highly selected PCS cohorts (34 and 35 patients, respectively) and requires confirmation in larger, unselected populations (17, 97). Identifying these patients early is clinically important: Kim et al. showed that patients with major PCS, defined by decreased mental alertness, hemiparesis involving at least two body compartments, aphasia/anarthria, or hemianopsia, presented with AVS/AIS in 24.2% of cases (32/132), and in most of these the vestibular symptoms were prodromal (23/32, 71.8%), preceding the major deficits by a median of 10.5 h. Isolated dizziness or vertigo may therefore be an early warning sign of impending neurological decline (100).

### 5.3 Ocular motor testing

Additional bedside ocular motor testing may be useful in these patients, although evidence remains limited. Smooth pursuit and saccadic abnormalities have been observed in stroke patients presenting without spontaneous nystagmus (108) and without marked gait or truncal instability (94), but the strongest diagnostic accuracy data for ocular motor testing still come from AVS cohorts. In a recent meta-analysis, the most sensitive bedside findings for a central cause of AVS were a normal h-HIT, grade 2 or 3 gait/truncal instability, and saccadic vertical pursuit, with sensitivities of 79.9, 80.8, and 84.3%, respectively, whereas other bedside tests, including the test of skew, direction-changing nystagmus on lateral gaze, and spontaneous vertical or torsional nystagmus, were considerably less sensitive when considered in isolation. Specificity for detecting a dangerous central cause of AVS, however, was high for all tests (85). There is also a plausible neuroanatomical basis for both tests. Pursuit may be impaired by lesions of the dorsolateral pons, the cerebellar peduncles, or the cerebellum itself, so that PICA- and AICA-territory infarcts may produce detectable pursuit abnormalities (109). Saccadic abnormalities may be more precisely localizing. Lateral medullary infarction (Wallenberg syndrome) may cause ocular lateral deviation (OLD) with ipsilesional hypermetric and contralesional hypometric saccades (110, 111), whereas lesions of the medial longitudinal fasciculus (MLF), paramedian pontine reticular formation (PPRF), and rostral interstitial nucleus of the MLF may produce internuclear ophthalmoplegia (INO), slowed horizontal or vertical saccades with or without gaze palsy (112), or an ocular tilt reaction (113).

## 6 Episodic isolated dizziness and posterior circulation TIA

The approaches described so far require the patient to be symptomatic when examined. This is not the case in patients whose dizziness or vertigo occurs in discrete attacks, typically lasting minutes, and who are free of symptoms and signs by the time they are seen. Such attacks meet neither the AVS nor the AIS definition and may represent a transient ischemic attack of the posterior circulation. In the ATTEST framework, these patients belong to the episodic spontaneous category, in which the differential diagnosis includes vestibular migraine (VM), Menière disease and TIA (71, 72). Among patients admitted for a sudden isolated vertigo or dizziness attack, recent infarction was found in 11.3%, mostly small cerebellar lesions (30). Recognition is difficult because the examination is often normal between attacks and early imaging is frequently negative (31), so a normal examination does not exclude a vascular cause. The vascular risk extends beyond the attack itself, as patients hospitalized with isolated vertigo carried a 3.01-fold risk of stroke over the following 4 years (34). A comparable warning function has been described for persistent presentations, where isolated dizziness or vertigo preceded the major deficits in 17.4% of patients with major PCS (23/132), usually within the first 24 h (100). In patients with episodic isolated dizziness or vertigo and vascular risk factors, vascular imaging of the posterior circulation and cardiovascular risk stratification should therefore be considered.

## 7 When bedside assessment and imaging remain inconclusive

Three situations should be distinguished when the work-up remains inconclusive. First, in HINTS(+)-eligible patients examined by trained clinicians, a peripheral pattern substantially lowers the probability of PCS (85, 87), the residual risk lies mainly with less experienced examiners, in which case quantitative testing such as vHIT/vHINTS may help where available (90–92, 114). Second, in patients without nystagmus, a benign bedside impression is less reassuring. Gait and truncal instability assessment and the STANDING algorithm retain moderate sensitivity for central causes (102, 105, 106), and the subgroup with neither nystagmus nor marked instability remains insufficiently captured (17, 97). Third, when the bedside examination suggests PCS but early DWI-MRI and vascular imaging are normal, a false-negative scan must be considered, particularly for small infarcts and early imaging (42, 68). Repeat MRI after 3–14 days is then the appropriate confirmatory step (70, 97), and admission or observation is warranted in patients with high-risk features (57, 70). Whether otherwise eligible patients with AVS/AIS and suspected DWI-negative PCS should receive acute reperfusion therapy remains an individual decision, as the available evidence is insufficient for a firm recommendation (98, 101). Discharged patients should receive explicit instructions, and where risk factors accumulate, early follow-up including vascular imaging (70). Figure 1 summarizes the proposed diagnostic approach.

**Figure 1:**
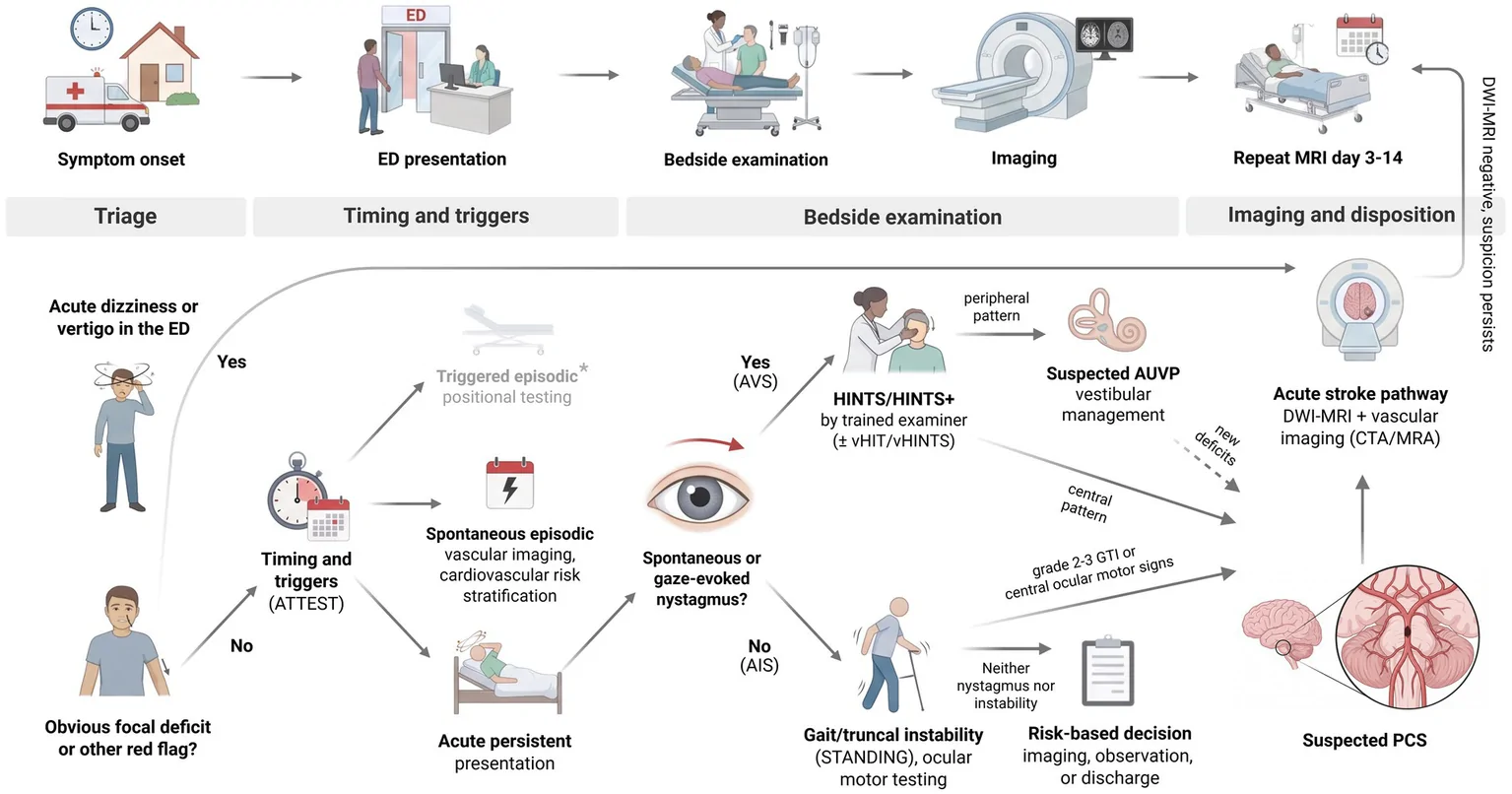
Proposed diagnostic approach to acute dizziness and vertigo when posterior circulation stroke (PCS) is suspected. The upper band shows the stages of the acute care pathway from symptom onset to repeat imaging. The algorithm below follows the same sequence from triage to imaging and disposition. Patients with obvious focal neurological deficits or other red flags enter the acute stroke pathway directly. All other patients are triaged by timing and triggers (ATTEST). Triggered episodic vestibular symptoms prompt positional testing, spontaneous episodic symptoms prompt evaluation for transient ischemic attack (TIA) including vascular imaging and cardiovascular risk stratification, and acute persistent presentations are stratified by the presence of spontaneous or gaze-evoked nystagmus. Patients with nystagmus undergo HINTS/HINTS+ performed by an at least moderately trained examiner, patients without nystagmus undergo graded gait and truncal instability assessment and ocular motor testing. A central pattern, grade 2–3 gait or truncal instability (GTI) or central ocular motor signs lead to DWI-MRI and vascular imaging. A peripheral pattern lowers the probability of PCS and supports vestibular management, but does not exclude it, and patients with an atypical course or new deficits are referred for imaging (dashed arrow). Where neither nystagmus nor marked instability is present, disposition follows a risk-based decision between imaging, observation and discharge with return advice. When clinical suspicion persists despite negative imaging, DWI-MRI is repeated after 3–14 days, and observation or admission is warranted in patients with high-risk features. The diagnostic categories shown are working diagnoses rather than confirmed ones. *Shown for completeness of the ATTEST triage, triggered episodic vestibular symptoms are not discussed further in this review. AIS, acute imbalance syndrome; ATTEST, Associated symptoms, Timing, Triggers, Examination Signs, and Testing; AUVP, acute unilateral vestibulopathy; AVS, acute vestibular syndrome; CTA, computed tomography angiography; DWI-MRI, diffusion-weighted magnetic resonance imaging; GTI, graded gait and truncal instability; HINTS, Head Impulse, Nystagmus, and Test of Skew; MRA, magnetic resonance angiography; PCS, posterior circulation stroke; STANDING, SponTAneous Nystagmus, Direction, head Impulse test, standiNG; TIA, transient ischemic attack; vHINTS, video-oculography HINTS; vHIT, video head impulse testing. Created in BioRender. Seetge, J. (2026) https://BioRender.com/zbegyh0.

## 8 Discussion

Diagnosing PCS in patients with acute dizziness and vertigo remains important but difficult. These patients are at risk of both underdiagnosis and misdiagnosis, which may lead to delays in stroke care and avoidable resource utilization, partly due to a continued focus on symptom quality, limited use of structured bedside ocular motor/balance assessments, and overreliance on neuroimaging.

None of the available approaches closes this gap on its own. Prehospital scales gain sensitivity for PCS only at the cost of considerable overtriage, in-hospital severity scales underrepresent posterior circulation deficits, and not every patient is eligible for HINTS/HINTS+. NCCT remains the primary imaging modality in about half of these patients despite its low sensitivity in this presentation, and even early DWI-MRI carries a relevant false-negative rate for PCS. Alternative and complementary diagnostic approaches are therefore needed.

It remains debated whether the bedside examination should be expanded further, or whether it would be more effective to train clinicians in the approaches that already exist, given that the examiner background explains a considerable part of the variability in diagnostic accuracy. The concept of the AIS has been introduced to capture patients who present without nystagmus and therefore fall outside the HINTS(+)-based algorithms. For this group, graded gait and truncal instability rating/the STANDING algorithm has been proposed, but the evidence remains heterogeneous and is derived largely from selected cohorts. A clinically important subgroup remains uncovered by this approach as well, namely patients with isolated dizziness or vertigo who have neither nystagmus nor marked gait or truncal instability.

Future work should therefore address the prospective validation of integrated bedside algorithms in unselected ED populations including patients without nystagmus, the systematic evaluation of training interventions, and diagnostic pathways for patients in whom bedside assessment and subsequent imaging remain inconclusive. Given that the most widely cited guidance for the emergency evaluation of acute dizziness and vertigo is still based on expert consensus rather than randomized evidence, prospective trials comparing structured diagnostic pathways with usual care would be of value. A standardized, evidence-informed diagnostic protocol that prioritizes a timing-and-triggers approach and includes a focused ocular motor examination performed by trained clinicians may help reduce diagnostic errors in this patient population.

## Glossary

ACS: anterior circulation stroke
AICA: anterior inferior cerebellar artery
AIS: acute imbalance syndrome
ATA: acute truncal ataxia
ATTEST: Associated symptoms, Timing, Triggers, Examination Signs, and Testing
AUVP: acute unilateral vestibulopathy
AVS: acute vestibular syndrome
BE-FAST: Balance, Eyes, Face, Arm, Speech, Time
CI: confidence interval
CT: computed tomography
CTP: computed tomography perfusion
DEFENSIVE: DisEquilibrium, Floating sEnsation, Non-Specific dizziness, Imbalance, and VErtigo
DWI-MRI: diffusion-weighted magnetic resonance imaging
ED: emergency department
e-NIHSS: expanded National Institutes of Health Stroke Scale
FAST: Face, Arms, Speech, Time
FAST4D: FAST extended with Diplopic images, Deficit in the field of vision, Dizziness/vertigo, and Dysmetria/ataxia
GRACE-3: Guidelines for Reasonable and Appropriate Care in the Emergency Department
GTI: graded gait and truncal instability
h-HIT: horizontal head impulse test
HINTS: Head Impulse, Nystagmus, and Test of Skew
HINTS+: HINTS plus bedside hearing assessment
INO: internuclear ophthalmoplegia
IVBSS: Israeli Vertebrobasilar Stroke Scale
MLF: medial longitudinal fasciculus
MRI: magnetic resonance imaging
MS: multiple sclerosis
NCCT: non-contrast computed tomography
NIHSS: National Institutes of Health Stroke Scale
OLD: ocular lateral deviation
OR: odds ratio
PCS: posterior circulation stroke
PICA: posterior inferior cerebellar artery
PPRF: paramedian pontine reticular formation
SCA: superior cerebellar artery
STANDING: SponTAneous Nystagmus, Direction, head Impulse test, standiNG
TIA: transient ischemic attack
TiTrATE: Timing, Triggers, and Targeted Examination
vHINTS: video-oculography HINTS
vHIT: video head impulse testing
VOR: vestibulo-ocular reflex
