# Supplementary material for: Diagnostic challenges in posterior circulation ischemic stroke presenting with acute dizziness and vertigo
Source: Front Neurol. 2026 Sep 24;17:1960712. doi: 10.3389/fneur.2026.1960712 (PMC13647463; doi:10.3389/fneur.2026.1960712)
Supplement: Supplementary file 1 [file Supplementary_file_1.docx]

**Supplementary Material**

Search terms used for the literature search

Rather than applying a single Boolean search string, terms were searched iteratively in the thematic groups listed below. Titles and abstracts of the retrieved records were screened for relevance, and the reference lists of relevant publications were hand-searched for additional literature.

## Group 1 - Stroke entity and vascular territory

posterior circulation stroke; vertebrobasilar stroke; vertebrobasilar ischemia; posterior fossa infarction; brainstem infarction; lateral medullary infarction; cerebellar infarction; nodulus infarction; cerebellar tonsil infarction; superior cerebellar artery; anterior inferior cerebellar artery; posterior inferior cerebellar artery; posterior cerebral artery

## Group 2 - Clinical presentation

dizziness; acute dizziness; isolated dizziness; vertigo; isolated vertigo; episodic isolated vertigo; imbalance; unsteadiness; acute vestibular syndrome; acute imbalance syndrome; acute unilateral vestibulopathy; vestibular neuritis; benign paroxysmal positional vertigo; vestibular migraine; sudden hearing loss; classification of vestibular symptoms; transient ischemic attack; episodic vestibular syndrome

## Group 3 - Diagnostic setting, diagnostic error and clinician factors

emergency department; prehospital assessment; triage; misdiagnosis; diagnostic error; missed stroke; stroke mimic; stroke chameleon; symptom quality; diagnostic reasoning; physician survey

## Group 4 - Bedside assessment and neuroimaging

bedside examination; ocular motor examination; nystagmus; spontaneous nystagmus; gaze-evoked nystagmus; direction-changing nystagmus; downbeat nystagmus; fixation suppression; head impulse test; video head impulse test; vestibulo-ocular reflex gain; skew deviation; ocular tilt reaction; ocular lateral deviation; smooth pursuit; saccades; saccadic dysmetria; internuclear ophthalmoplegia; gaze palsy; medial longitudinal fasciculus; paramedian pontine reticular formation; gait and truncal instability; truncal ataxia; postural instability; HINTS; HINTS plus; STANDING; TiTrATE; ATTEST; GRACE-3; prehospital stroke scale; FAST; BE-FAST; FAST4D; NIHSS; expanded NIHSS; posterior NIHSS; vertebrobasilar stroke scale; clinical risk score; neuroimaging; computed tomography; computed tomography perfusion; magnetic resonance imaging; diffusion-weighted imaging; DWI-negative stroke; vertebral artery dissection; computed tomography angiography; magnetic resonance angiography; Doppler ultrasound

## Group 5 - Acute treatment and outcome

intravenous thrombolysis; reperfusion therapy; door-to-needle time; stroke risk; recurrent stroke; vascular events; prognosis

Abbreviations: ATTEST = Associated symptoms, Timing, Triggers, Examination Signs, and Testing; BE-FAST = Balance, Eyes, Face, Arm, Speech, Time; DWI = diffusion-weighted imaging; FAST = Face, Arms, Speech, Time; FAST4D = FAST extended with Diplopic images, Deficit in the field of vision, Dizziness/vertigo, and Dysmetria/ataxia; GRACE-3 = Guidelines for Reasonable and Appropriate Care in the Emergency Department; HINTS = Head Impulse, Nystagmus, and Test of Skew; NIHSS = National Institutes of Health Stroke Scale; STANDING = SponTAneous Nystagmus, Direction, head Impulse test, standiNG; TiTrATE = Timing, Triggers, and Targeted Examination.
